# Primary abdominal cocoon with cryptorchidism: a case report

**DOI:** 10.1186/s12876-020-01466-x

**Published:** 2020-10-14

**Authors:** Wei-Jie Song, Xin-Yi Liu, Galal Abdullah Ali Saad, Aawrish Khan, Kai-Yan Yang, Yi Zhang, Jian-Ye Liu, Le-Ye He

**Affiliations:** 1 Department of Urology, Central South University, The Third Xiangya Hospital, No. 138 Tongzipo Road, Changsha City, 410013 Hunan Province China; 2 Institute of Prostate Disease, Central South University, Changsha, Hunan China; 3 Department of Obstetrics and Gynecology, Central South University, The Third Xiangya Hospital, Changsha, Hunan China; 4 Department of Gastrointestinal Surgery, Central South University, The Third Xiangya Hospital, Changsha, Hunan China

**Keywords:** Cryptorchidism, Abdominal cocoon syndrome, Cryptorchid surgery, Prognosis, Case report

## Abstract

**Background:**

We report a rare case of primary abdominal cocoon with bilateral cryptorchidism.

**Case presentation:**

The patient had a history of laparoscopic surgery for bilateral cryptorchidism 6 years earlier. He was admitted to the hospital again due to intestinal obstruction. Surgery was performed on the patient after the failure of conservative treatment. The patient was diagnosed with primary abdominal cocoon. Instead of the greater omentum, many cocoon-like tissues surrounding the bowel were seen during operation. Abdominal surgery can increase the risk of intestinal adhesion, which is one of the main causes of intestinal obstruction, especially in patients with abdominal cocoon. We hypothesize that the surgery 6 years earlier to address transabdominal bilateral cryptorchidism accelerated the patient’s intestinal obstruction.

**Conclusion:**

This case implies that it is important for urologists to evaluate whether their patients exhibit abdominal cocoon before cryptorchidism surgery, to choose better surgical methods and reduce the risks of poor prognosis.

## Background

Abdominal cocoon syndrome (ACS), a rare disease of the abdominal organs, is characterized by a dense, pale abnormal fibrous collagen membrane wrapping part or all of the intestine like a cocoon, usually known as primary or secondary abdominal cocoon, as distinguished later in this report. Cryptorchidism is a congenital testicular malformation that can be divided into unilateral or bilateral cryptorchidism, appearing as a missing testicle in the affected lateral scrotum [1]. Common symptoms of ACS include abdominal pain, bloating, vomiting, and abdominal masses. It has been reported that tuberculosis may be related to the formation of abdominal cocoon [2]. However, coexistence of ACS with cryptorchidism is very rare, and to our knowledge, there have been only two case reports of ACS with unilateral cryptorchidism. The exact causes of this disease are unknown.

## Case presentation

**Fig. 1:**
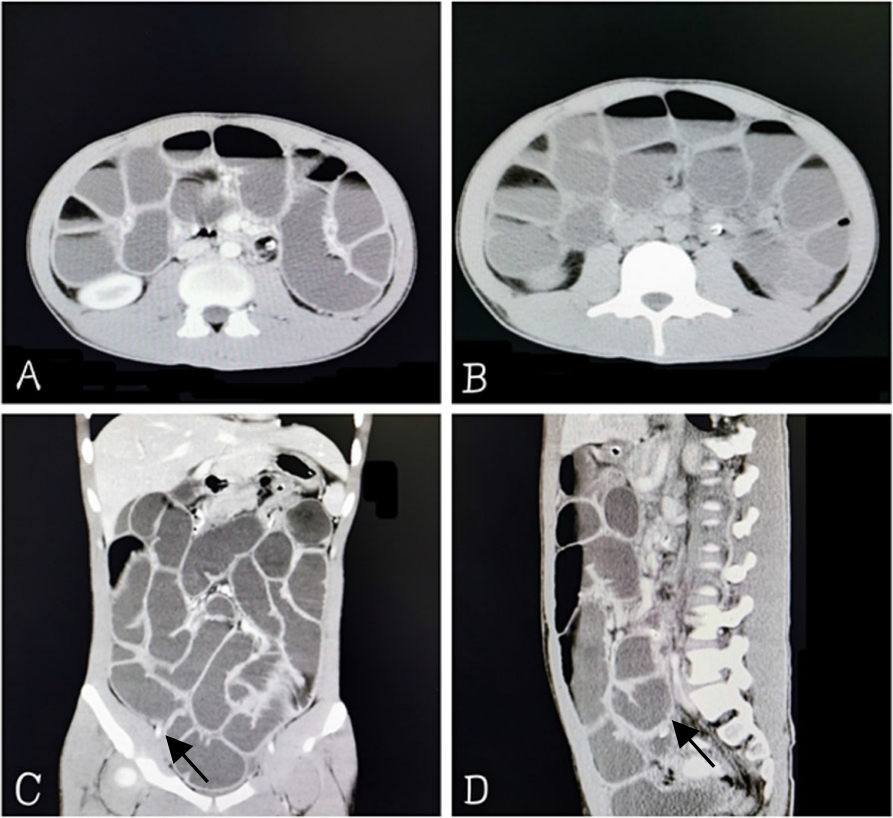
Images from abdomen CT. Panels **a** and **b**: CT of the abdominal transverse plane. Intestinal dilation, gas and fluid can be seen in the intestine. Panel **c**: CT of the anterior abdomen, sagittal. A cystic membrane is visible around the intestine, and the intestinal wall is abnormally thickened (shown with arrow). Panel **d**: CT of the abdominal sagittal plane. A fibrous membrane is visible around the intestine (shown with arrow)

In this case, a 19-year-old male patient had been diagnosed with bilateral cryptorchidism for bilateral scrotal emptiness 6 years earlier. The left testicle was removed by laparoscopic surgery, and the right testicle was lowered and fixed. The operation was successful. In December 2019, the patient was re-admitted to the hospital, due to abdominal pain accompanied by nausea and vomiting for 3 days. By examination, the abdomen was swollen, and tenderness but no rebound pain was reported around the umbilicus. The abdominal muscles were slightly tense, and bowel sounds were active approximately 6–7 times/minute. An abdominal plain film indicated that gases and liquids had accumulated in the intestine, and a visible liquid-gas level could be seen. Enhanced computed tomography (CT) of the abdomen suggested the possibility of mechanical obstruction at the level of the distal ileum. A routine blood test reported 16.63 * 10 ^ 9 / L white blood cells, 0.73 * 10 ^ 9 / L monocytes, 84.3% neutrophil percentage, and 14.02 * 10 ^ 9 / L neutrophils, suggesting the presence of infection. Based on the examination results in conjunction with the patient’s existing symptoms, signs, and previous medical history, the initial diagnosis was adhesion intestinal obstruction related to laparoscopic cryptorchidism surgery. An intestinal obstruction catheter was installed under emergency gastroscopy for conventional conservative treatment. After 2 weeks, the patient’s abdominal symptoms had improved significantly. Peristalsis and (minimal) defecation resumed, but the intestinal obstruction catheter remained at 170 cm without improvement. A total gastrointestinal angiography was performed to reconfirm the intestinal obstruction, and the results still indicated incomplete distal small bowel obstruction. Thus, conservative treatment was continued. Approximately 3 days later, the patient reported a sudden return of abdominal tenderness and intolerable pain. Reexamination of the whole abdominal CT showed that the intestinal effusion was more dilated than before, the gas-liquid level was elevated, the intestinal lumen had expanded and the intestinal obstruction was exacerbated, appearing as abnormal thickening of the small intestinal wall, as shown in Fig. 1.

**Fig. 2:**
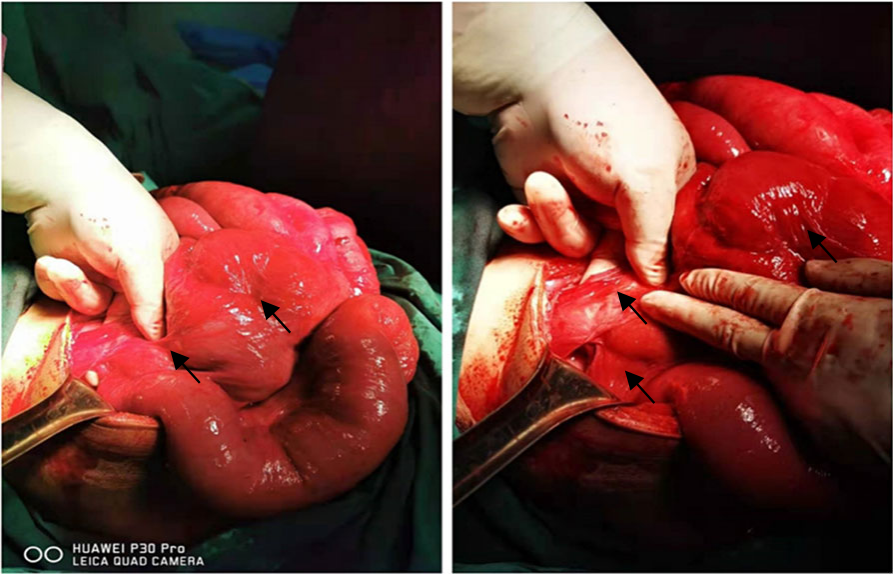
Photographs of the surgery scene. No omentum majus tissue was found in the abdominal cavity. Natural fibrous tissue can be seen on the surface of the intestine, widely adhered to form an adhesion band (shown with arrows)

Conservative treatment was ceased, and laparotomy was performed immediately to prevent intestinal necrosis. No greater omentum was found during surgery, with fibrous tissue seen instead on the surface of the intestine, which was tightly attached and wrapped around the intestine (see Fig. 2). In consideration of the patient’s history of cryptorchidism, he was diagnosed with congenital cryptorchidism with primary abdominal cocoon. The patient’s intestine could still be seen under the capsule, as the cocoon membrane is thin, although the extensive adhesions form fibrous bands. Intestinal adhesion release, intestinal decompression and intestinal alignment were performed; the operation went smoothly. On the 3rd day after surgery, the patient was able to defecate, and no notable discomfort such as bloating, pain, nausea or vomiting was reported at the 10th day after surgery. Consequently, the abdominal drainage tube and intestinal obstruction tube were removed. Bowel obstruction of the patient has not recurred and is still being followed up.

## Discussion and conclusion

Cryptorchidism is a common disease in urology, and patients over 1 year of age require surgical treatment. Low cryptorchidism, where a testis can be palpated, can be addressed by open surgery in the groin or scrotum or by laparoscopic surgery [3]. For high cryptorchidism, traditional surgical operation is more difficult, as the testicles cannot be easily palpated. In recent years, laparoscopic cryptorchidism surgery has been widely recommended by doctors and preferred by patients for its many advantages [4, 5]. Compared with cryptorchidism, ACS is an extremely rare disease that can be divided into two types: primary and secondary. Primary abdominal cocoon is a congenital disease that may be caused by congenital developmental abnormalities and/or autoimmune reactions. Secondary cocoon is more common in patients with a history of abdominal surgery or certain drug consumption for a long time [6]. A typical anatomical feature of ACS is that the surface of the intestine is wrapped with cocoon-like fibrous tissue [7]. Unless intestinal obstruction frequently recurs, conservative treatment is recommended for ACS patients. It is possible for patients with mild ACS to live a healthy life with the cocoon [8].

However, transabdominal surgery necessitates caution for ACS patients. Pulling, separating, and suturing to the intestine during abdominal surgery may affect the continuity and integrity of the peritoneal epithelium, causing inflammatory reactions in the peritoneum and/or intestinal serosa and leading to intestinal adhesions. Moreover, increased postoperative inflammation brings a higher risk of intestinal adhesions [9]. As a result, transabdominal surgery can easily induce intestinal obstruction, especially for patients with ACS. The patient in our case exhibited primary abdominal cocoon coexisting with bilateral cryptorchidism. His intestinal obstruction caused by severe intestinal adhesions appeared when he was only 19 years old, which may have been related to the cryptorchidism laparoscopy surgery 6 years earlier. The laparoscopic surgery, in our opinion, accelerated the patient’s complications associated with primary abdominal cocoon. If the patient had not undergone laparoscopy for cryptorchidism, he might not have had ACS bowel obstruction at such a young age.

Thus, we suggest that urologists consider the possibility of coexisting ACS before performing cryptorchidism surgery. If a patient is diagnosed with ACS with cryptorchidism, transabdominal surgery should be avoided if possible to reduce the risk of complications.

## Data Availability

Data sharing is not applicable to this article, as no datasets were generated or analyzed during the current study.

## Abbreviations

ACS: Abdominal cocoon syndrome
CT: Computed tomography
